# Effects of Upregulation of TNFAIP3 on Diabetic Neuropathic Pain in Mice

**DOI:** 10.1155/2021/3470950

**Published:** 2021-11-22

**Authors:** Yang Liu, Jinhe Li, Hongbo Yao, Meng Zhang, Jie Lian, Haiyan Zhang, Keshuang Zhang, Danyang Liu, Jiwei Chen, Yuejing Wang, Yin Gao

**Affiliations:** ^1^Department of General Surgery, Qiqihar First Hospital, Qiqihar 161006, China; ^2^Department of Embryology, Qiqihar Medical College, Qiqihar 161006, China; ^3^Department of Anatomy, Qiqihar Medical College, Qiqihar 161006, China

## Abstract

Globally, diabetes has assumed epidemic proportions with the neuropathic complications attributed to the malady emerging as a substantial burden on patients and society. DNP has greatly affected the daily life of patients, the effect of traditional treatment methods is not ideal, and it is easy to produce drug resistance. This work is aimed at scrutinizing the effect of upregulating the expression of TNFAIP3 on diabetic neuralgia in mice. This work entailed ascertaining the effects of TNFAIP3 on a murine DNP system. This inspired us to observe the analgesic effect via high expression of lentivirus-mediated TNFAIP3 by intrathecal injection in the animal model to explore its regulatory impacts, symptom relief, and mechanistic role in pain. The results displayed an attenuation of hind paw pain hypersensitivity by LV-TNFAIP3 in the animals. The spinal cord and dorsal root ganglion of mice with neuropathic pain displayed an evident dip in TNFAIP3. Inhibition of the ERK/NF-*κ*B signaling pathway employing LV-TNFAIP3 conspicuously suppressed this pathway while the diabetic pain hypersensitivity was quelled. This effect was also seen with insulin treatment evidently. In conclusion, according to the above analyses, the interaction between DNP and extracellular signal-regulated kinase signal transduction pathway is one of the key factors of pathogenesis.

## 1. Introduction

Globally, diabetes has assumed epidemic proportions with the neuropathic complications attributed to the malady emerging as a substantial burden on patients and society. Neuropathic pain is caused by diseases of the body. When neuropathic pain is diagnosed, damage to the sensory nervous system is seen with DNP impacting up to 8% of the population [1]. The problem of DNP is its severity with effective treatments yet to see the light of the day. Diabetes is mainly divided into two types: type 1 diabetes and type 2 diabetes. DNP is a ubiquitous complication of diabetes. The lifetime incidence of DNP in type 2 diabetics is 45~70% [2, 3]. The early symptom of DNP is sensory nerve disorder with patients usually experiencing sensory symptoms, such as pain, tingling, paresthesia, and numbness [4, 5].

DNP has greatly affected the daily life of patients, the effect of traditional treatment methods is not ideal, and it is easy to produce drug resistance [6, 7]. Therefore, in this study, gene therapy is selected to alleviate the pain. One of the key problems of gene therapy is to select the appropriate vector to make the treatment gene safely and efficiently transferred into the target cells. This work is aimed at scrutinizing the effect of upregulating the expression of TNFAIP3 on diabetic neuralgia in mice. This entails an intrathecal injection of lentiviral-based TNFAIP3 to upregulate the expression of TNFAIP3 in a DNP mouse model along with the involvement of the ERK/NF-*κ*B signaling pathway. Further, pain in the spinal cord and dorsal root ganglia (DRGs) was also explored.

## 2. Research Design and Methods

### 2.1. Murine DNP Model Generation

All experiments were approved by the Animal Protection and Use Committee of Qiqihar Medical University. C57/BL6 mice (5-7-week-old: 16-25 g in weight) were housed in cages (4/cage) in a 12 hour-12 hour light-dark cycle at 25 ± 1°C.The mice were allowed to drink tap water and eat standard laboratory food at will. Following a 28-day high-sugar and high-fat feed, assays were initiated. Fasting ensued (inclusive of water) for more than 12 hours. Diabetes induction entailed the intraperitoneal injection of 30 mg/kg streptozotocin (STZ), a total of 2 times, 3 days apart that was ascertained by a fasting blood glucose level > 11.1 mol/L. This was followed by an instantaneous screening of mice manifesting pain complications that were indicative of the successfully establishing the DNP model. Screened DNP models were further used in the study.

### 2.2. Lentiviral Vector Intrathecal Injection

Following an intraperitoneal injection with 10% chloral hydrate to anesthetize an animal, the L4-6 lumbar spinous process was secured by one hand, and the skin pierced with a thick needle to pierce the subarachnoid with a 2-5 ul micro syringe employing the other hand. Following penetration of the cavity, the needle was inserted until the typical rat tail quivers and shakes indicative of successful puncture and intrathecal Lv-TNFAIP3 injection.

### 2.3. Measurement of Hind Paw Withdrawal Threshold

Prior to commencing the ascertaining of this threshold, placing the animals for one week in the test environment was done to facilitate habituation to the surrounding. Following the one-week habituation, the experiment entailed two approaches: paw withdrawal threshold (PWT) and thermal paw withdrawal latency (PWL). The former is the response to the Von Frey filament stimulation based on radiant heat application to the plantar surface of hind paw while the latter is in response to the Von Frey filament stimulation. All experiments were on the lines of earlier research [8]. All behavioral experiments were started employing blinded conditions.

### 2.4. Western Blotting Analysis

This was done to quantify TNFAIP3, ERK2, ERK1/2, MAPKp38, NF-KB, and p65 levels in L4-L6 of the spinal cord and DRGs [9]. The DPN mice and controls were the sources of tissues with the former entailing with or without insulin treatment-based processing. Following LV-TNFAIP3 treatment and siRNA treatment, TNFAIP3 (1 : 500, Abcam, USA) and antibodies against ERK2, ERK1/2, MAPKp38, NF-KB, p65 antibodies (1 : 800, Abcam, USA), and corresponding horseradish peroxidase-conjugated secondary anti-rat antibodies (at 1 : 1000 dilution) were employed for the assay.

### 2.5. Toluidine Blue Staining and Immunohistochemical Staining

This entailed the following: once the animals were anesthetized, 4% paraformaldehyde-based perfusion was done followed by sampling. The spinal cord and DRGs were embedded in paraffin and sliced as 10 micrometers thick sections employing a paraffin microtome. Subsequent toluidine blue staining entailed the use of the staining kit adhering to the prescribed protocols. Immunohistochemistry employed the use of TNFAIP3 (Rabbit, 1 : 200, Abcam, USA) followed by assessing and imaging in a confocal microscope.

### 2.6. Data Analysis

This entailed the use of SPSS statistics 17.0 for results expressed as the mean ± SEM. Two sample *t*-test was employed for significance determination. Application ImageJ software analysis (NIH, Bethesda, Maryland, USA) was employed for Western blot results for both intra- and intergroup analyses. Significance across groups was at a *P* value less than 0.05.

## 3. Results

**Figure 1:**
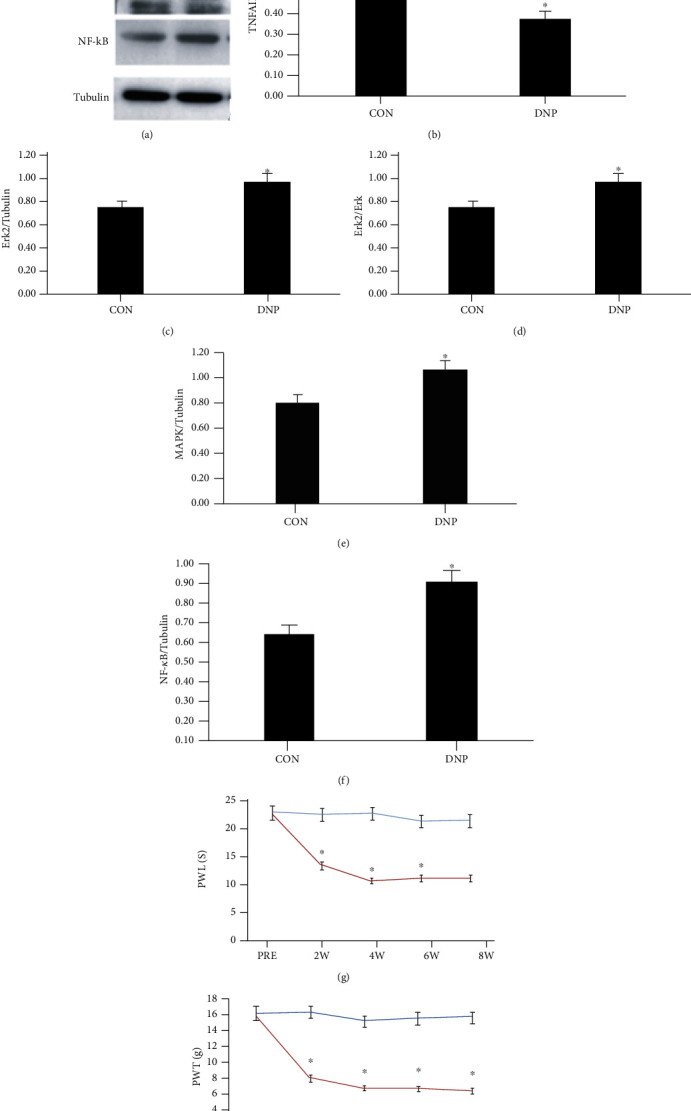
Changes of expression of TNFAIP3, ERK2, MAPK, and NF KB and pain threshold in Con and DNP groups. One-way ANOVA revealed significant variations across the groups (*P* < 0.05). The results of Western blot are as shown in (a). (b)–(f) are the expression of TNFAIP3, ERK2, MAPK, and NF-*κ*B, respectively. (g) and (h) are the results of PWL and PWT.

**Figure 2:**
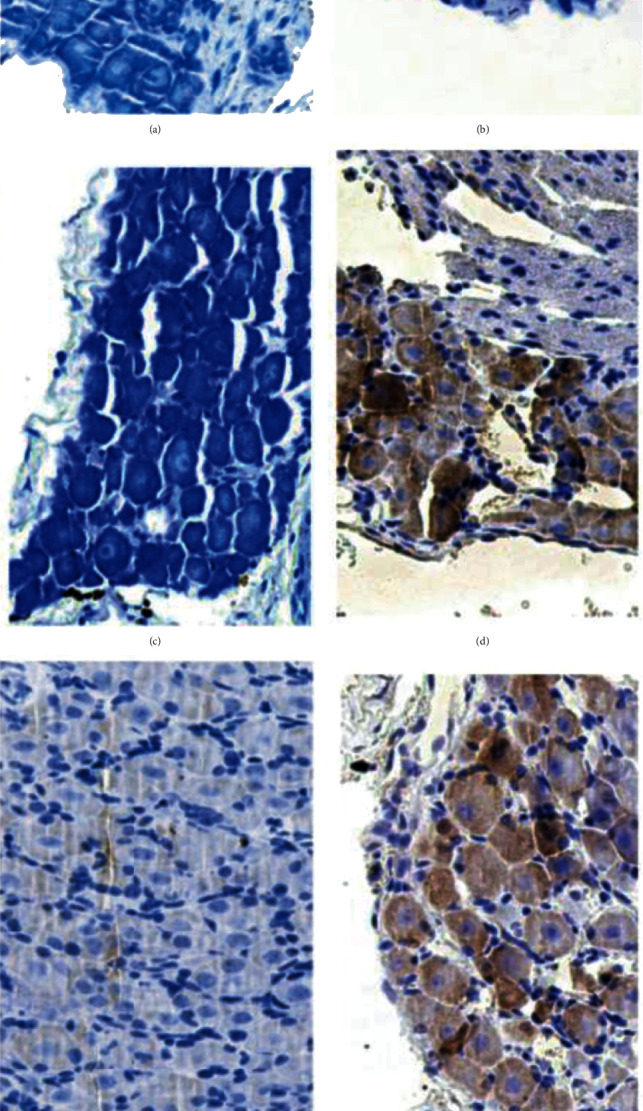
Morphological changes and TNFAIP3 expression in ganglion neurons in sham, DNPsiRNA, and DNP-TNFAIP3 groups. One-way ANOVA statistically significant variations (*P* < 0.05). The results of toluidine blue staining are as shown in (a)–(c) for sham, DNPsiRNA, and DNP-TNFAIP3 groups, respectively. The results of immunohistochemical staining are as shown in (d)–(f) for sham, DNPsiRNA, and DNP-TNFAIP3 groups, respectively. (g) is TNFAIP3 expression across the groups.

**Figure 3:**
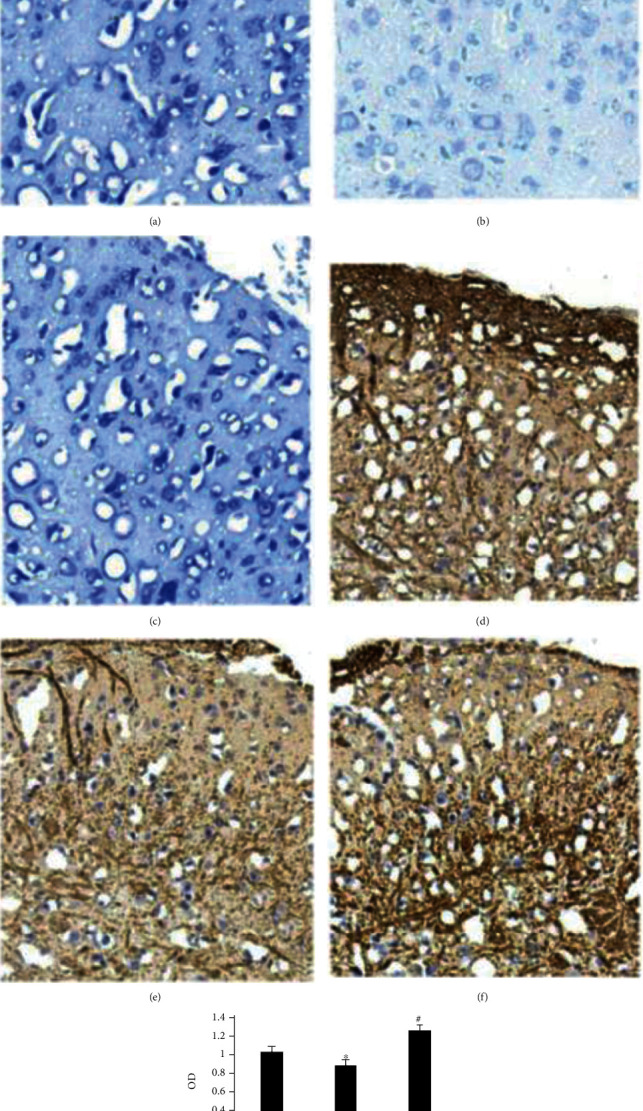
Morphological changes and TNFAIP3 expression in the posterior horn of the spinal cord in sham, DNPsiRNA, and DNP-TNFAIP3 groups. One-way ANOVA revealed statistically significant variations (*P* < 0.05); the results of toluidine blue staining are as shown in (a)–(c): (a) is sham; (b) is DNPsiRNA group; (c) is DNP-TNFAIP3 group. The results of Immunohistochemical staining are as shown in (d)–(f): (d) is sham, (e) is DNPsiRNA group; (f) is DNP-TNFAIP3 group; (g) is TNFAIP3 expression across groups.

**Figure 4:**
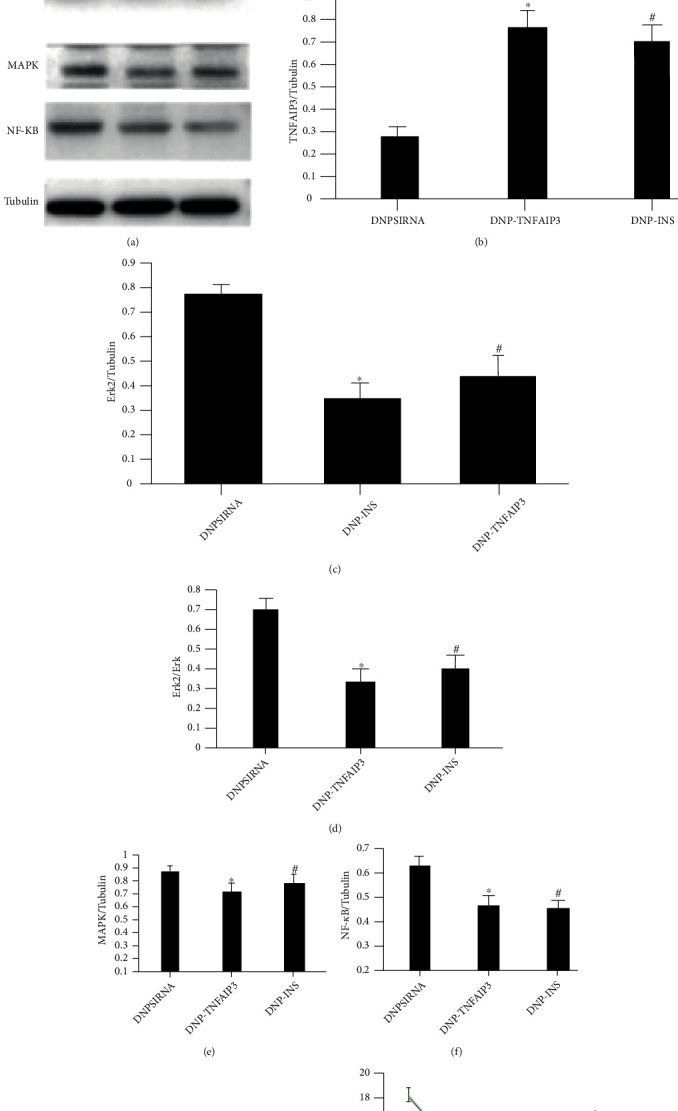
Expression changes of TNFAIP3, ERK2, MAPK, and NF KB in the posterior horn of spinal cord and pain threshold in sham, DNPsiRNA, and DNP-TNFAIP3 groups. One-way ANOVA revealed statistically significant alterations (*P* < 0.05). The results of Western blot are as shown in (a). (b)–(f) are TNFAIP3 expression, ERK2 expression, MAPK expression, and NF-*κ*B expression. (g) and (h) are the results of PWL and PWT.

All results are as shown in Figures 1–4.

In Figure 1, the results of Western blot were shown in (a). (b)–(f) are the analyses. (b) shows conspicuously lower TNFAIP3 level in DNP vs. sham. (c) and (d) show the conspicuous increase in ERK2 and MAPK proteins, respectively, in DNP vs. sham. Evidently higher NF-*κ*B in DNP vs. sham. (g) and (k) show the mechanical pain threshold and thermal pain threshold. The pain threshold in the DNP group was significantly decreased at the 2nd, 4th, 6th, and 8th week vs. sham (*P* < 0.05 for all tests).

In Figure 2, results of toluidine blue staining of mouse ganglion tissue are shown in (a)–(c).

(a) is Con group, (b) is the DNP siRNA group, and (c) is the DNP-TNFAIP3 group. The contour of ganglion neurons of the DNPsiRNA group is not clear vs. sham: Nissl body is not clear, the nucleolus is not obvious, and cell survival rate is low. However, the sham and DNP-TNFAIP3 groups display a clear contour and Nissl body with an obvious nucleolus.

Immunohistochemical staining results of mouse ganglion tissues are shown in (d)–(f), (d) is sham group, (e) is the DNPsiRNA group, (f) is the DNP-TNFAIP3 group, and (g) is gray value analysis of each group. Lower TNFAIP3 in the DNP siRNA set vs. sham while the DNP-TNFAIP3 group displayed an evident increase in TNFAIP3 (^∗^ and ^#^*P* < 0.05).

In Figure 3, the results of toluidine blue staining of mouse spinal cord tissue are shown below.

(a) (20 *μ*m) is sham, (b) (20 *μ*m) is the DNP siRNA group, and (c) (20 *μ*m) is the DNP-TNFAIP3 group. Compared with sham, the outlines of ganglion neurons, Nissl body, and nucleolus in the DNP siRNA group are not clear; however, they are clear in sham and DNP-TNFAIP3 groups.

Immunohistochemical staining results of spinal cord tissue of mice are shown in (d)–(f).

(d) (20 *μ*m) is sham, (e) (20 *μ*m) is the DNP siRNA group, (f) (20 *μ*m) is the DNP-TNFAIP3 group, and (g) is the gray value analysis of each group. Lowered TNFAIP3 in the DNPsiRNA group vs. sham (^∗^*P* < 0.05); evidently lower TNFAIP3 in DNP-TNFAIP3 vs. DNP siRNA (^#^*P* < 0.05).

(6) Mechanical pain threshold and heat pain threshold changes and TNFAIP3 expression in the DNPsiRNA, DNP-TNFAIP3, and DNP INS groups

Figure 4 illustrates western blots of sham, DNPsiRNA, and DNP-TNFAIP3 groups and PWT (G) and PWL (S).

Overall, these data are suggestive of insulin loss or/and hyperglycemia but not STZ neuronal toxicity [10] as the most putative reasons attributed to predominant biochemical aberrations observed in this work.

## 4. Discussion

Diabetes is one of the focal and vital underlying disease and risk factors of ischemic stroke. Ischemic stroke patients with diabetes have more serious neurological deficits, slower recovery, worse intravenous thrombolysis effect, higher recurrence rate, and worse prognosis [11, 12]. The mechanism remains to be comprehended completely with a vital involvement of inflammation displayed by studies. After cerebral ischemia-reperfusion, inflammatory cell activation is followed by the release of proinflammatory factors such as TNF-*α* and IL-1*β*. Further adhesion, chemotaxis, and inflammatory cell activation result in a slew of factors as an inflammatory cascade to further damage the blood-brain barrier to cause ischemia-reperfusion injury via the NF-*κ*B pathway. This release of excitatory neurotransmitters directly or indirectly causes nerve damage [13, 14].

TNFAIP3, also known as zinc lipoprotein A20, is a member of the ubiquitin-editing enzyme family. It is both deubiquitinating and is an ubiquitin ligase. The vital involvement of this protein in regulating inflammatory signal transduction [15, 16] to negatively regulate the NF-*κ*B pathway via feedback and inhibit NF-*κ*B pathway activation via several inflammatory signals has been shown [17–20]. It can inhibit the transcription of downstream inflammatory factors and has a strong anti-inflammatory effect. Research has pointed out to the alteration of this inflammation can lower the nerve damage caused by ischemia and reperfusion to thereby exert a protective effect on neurons. This inspired us to observe the analgesic effect of a lentivirus–based approach of high expression of TNFAIP3 via intrathecal injection into a diabetic neuropathic pain (DNP) mouse model. This was aimed at the impact of the significant upregulation of TNFAIP3 on DNP mice to further scrutinize the impact on DNP symptoms and unearth putative pain mechanisms. This work unveiled the crucial functioning of TNFAIP3 in DNP development.

Extracellular signal regulated kinase (ERK), a mitogen activated protein of the white kinase (MAPK) family, mediates the intracellular transduction of various signals [21, 22]. Elevated phosphorylation of this protein has been reported in different pain models. Given that murine hyperalgesia can be alleviated by blocking its upstream kinase, altering the activity can be linked to the transmission of deleterious stimulation and neurosensitivity. ERK activation is the key to signal transduction from the cell membrane surface receptor to the nucleus, with the involvement of upstream elements such as GTPase RAS, Raf-1, serine/threonine kinase, and bispecific kinase. ERK is crucially involved in the incidence and progression of pathological pain.

ERK includes two subtypes: extracellular signal-regulated kinase-1 (erk-1) and extracellular signal-regulated kinase-2 (ERK-2). Gene knockout experiments show that ERK-2 can completely replace the function of erk-1, but erk-1 cannot replace the function of ERK-2 [23–25]. Therefore, this pathway can be scrutinized by blocking ERK-2. In addition, gene regulation also focally involves the role of nuclear factor kappa B (NFkB). Its activation can delay the apoptosis of neutrophils, prolong their life cycle, and increase their number, thus, activating and producing a large number of inflammatory mediators and free radicals. In the MAPK family, the ERK1/2-NFkB pathway is involved in cell growth, development, proliferation, differentiation, and apoptosis.

Scholars corroborated the augmentation of A20 by E. phaseoloides (ES) to inhibit the phosphorylation of ERK1/2 and p38 and target the inflammatory response in collagen-induced arthritis (CIA) rats [26]. Therefore, this work entailed ascertaining the effects of TNFAIP3 (referred to as “A20”) on a murine DNP system. This inspired us to observe the analgesic effect via high expression of lentivirus-mediated TNFAIP3 by intrathecal injection in the animal model to explore its regulatory impacts, symptom relief, and mechanistic role in pain.

We demonstrate the impact of TNFAIP3 in murine DNP. In previous studies, we found that the expression of TNFAIP3 in a cerebral ischemia-reperfusion mouse model was significantly reduced. Two weeks after injection of LV-TNFAIP3, the neurological function score and cerebral infarction area were lowered indicative of the therapeutic effect of TNFAIP3 in mice. Western blot and immunohistochemical experiments highlighted evident restoration of TNFAIP3 protein after injection of LV-TNFAIP3 in the mouse system suggestive of the protective effect of TNFAIP3 on the brain tissue of the murine model of ischemia-reperfusion. This led to the question of the putative protective impact of LV-TNFAIP3 on DNP mice. We started a follow-up experiment with LV-TNFAIP3 injection in the DNP mice to show increased thermal pain threshold and mechanical pain threshold 2 weeks after injection that is suggestive of the utility of LV-TNFAIP3 to treat DNP mice.

The next step was the assaying of the putative mechanism of specific treatment. The results revealed conspicuous lowering of ERK/NF-KB pathway proteins in the spinal cord and DRGs of the DNP mice following the upregulation of TNFAIP3. This is suggestive of an inhibited ERK/NF-KB following TNFAIP3 protein expression upregulation. The following step entailed exploring the neuroprotective mechanism of TNFAIP3 on the mice. Observations unveiled an evident suppression of the ERK/NF-KB pathway proteins suggestive of inhibition of this cascade via TNFAIP3 upregulation.

Our results show no evident alteration of the mechanical pain threshold and thermal pain threshold of the DNP group against the sham group, before (pre) modeling. The threshold of the DNP animals dipped at 2, 4, 6, and 8 weeks after modeling. At the 8th week after modeling, the spinal cord tissues were sampled for tests. Western blot and immunohistochemistry revealed a conspicuously lower TNFAIP3 expression in the DNP group against the sham.

In order to further study whether upregulation of TNFAIP3 expression affects the MAPK signaling pathway, further research was done. The pain threshold reported in this work displayed no evident change in DNP-TNFAIP3 before model making (PRE) and the DNP-INS groups as opposed to the DNPsiRNA group. Both thresholds displayed a conspicuous increase at 2, 4, 6, and 8 weeks after modeling for the DNP-TNFAIP3 and DNP-INS groups as against the DNPsiRNA group. As earlier mentioned, analyses of the 8th-week spinal cord samples revealed evidently higher TNFAIP3 expression in DNP-TNFAIP3 and the DNP-INS groups compared to the DNPsiRNA group.

## 5. Conclusions

In conclusion, according to the above analyses, the interaction between DNP and extracellular signal-regulated kinase signal transduction pathway is one of the key factors of pathogenesis. Intrathecal injection of adenovirus carrying multiple genes can significantly improve the pain threshold of mice with neuropathic pain to thus quell pain with efficacy. However, reports of the effect of lentivirus-based TNFAIP3 expression augmentation on diabetic neuropathic pain are scarce. Therefore, this project intends to further take up TNFAIP3 as an effective target employing lentivirus as a vector to explore the effect and mechanism of upregulating TNFAIP3 on diabetic neuropathic pain.

## Data Availability

All data relevant to the study are included in the article.
